# Locus specific endogenous retroviral expression associated with Alzheimer’s disease

**DOI:** 10.3389/fnagi.2023.1186470

**Published:** 2023-07-06

**Authors:** Tyson Dawson, Uzma Rentia, Jessie Sanford, Carlos Cruchaga, John S. K. Kauwe, Keith A. Crandall

**Affiliations:** ^1^Computational Biology Institute, The George Washington University, Washington, DC, United States; ^2^Department of Biostatistics and Bioinformatics, Milken Institute School of Public Health, The George Washington University, Washington, DC, United States; ^3^Department of Psychiatry, Washington University School of Medicine, St. Louis, MO, United States; ^4^Department of Biology, Brigham Young University, Provo, UT, United States

**Keywords:** Alzheimer’s disease, HERV, endogenous retrovirus, RNA-Seq, gene expression

## Abstract

**Introduction:**

Human endogenous retroviruses (HERVs) are transcriptionally-active remnants of ancient retroviral infections that may play a role in Alzheimer’s disease.

**Methods:**

We combined two, publicly available RNA-Seq datasets with a third, novel dataset for a total cohort of 103 patients with Alzheimer’s disease and 45 healthy controls. We use telescope to perform HERV quantification for these samples and simultaneously perform gene expression analysis.

**Results:**

We identify differentially expressed genes and differentially expressed HERVs in Alzheimer’s disease patients. Differentially expressed HERVs are scattered throughout the genome; many of them are members of the HERV-K superfamily. A number of HERVs are correlated with the expression of dysregulated genes in Alzheimer’s and are physically proximal to genes which drive disease pathways.

**Discussion:**

Dysregulated expression of ancient retroviral insertions in the human genome are present in Alzheimer’s disease and show localization patterns that may explain how these elements drive pathogenic gene expression.

## 1. Introduction

Alzheimer’s disease is a chronic neurodegenerative disease and the leading cause of dementia (Alzheimer’s Association, 2010); its precise pathogenesis is uncertain but involves neuroinflammation (Heneka et al., 2015), extracellular neuritic plaques comprised of misfolded amyloid-β peptides (Tiraboschi et al., 2004), and intracellular neurofibrillary tangles comprised of the microtubule-associated protein tau (Brion, 1998). Tau-mediated mechanisms have been associated with loss of genomic stability in affected neurons (Madabhushi et al., 2014) and with the activation of human endogenous retroviruses (HERVs) (Guo et al., 2018).

Human endogenous retroviruses (HERVs) are a constituent part of the human genome and comprise ∼8% of all human DNA sequences (Hoyt et al., 2022). HERV insertion and replication events have happened throughout evolutionary history (Bannert and Kurth, 2006; Feschotte and Gilbert, 2012). Though most HERVs remain largely neutralized from mutations and epigenetic control mechanisms (Groh and Schotta, 2017), they still retain transcriptional activity (Seifarth et al., 2005; Pehrsson et al., 2019) and serve cell-type-specific regulatory functions (Stauffer et al., 2004; Ohnuki et al., 2014; Buzdin et al., 2017). Indeed, among all the transposable elements, HERVs are the most enriched for apparent regulatory functions (Jacques et al., 2013; Sundaram et al., 2014). Systematic characterizations of the regulatory functions of HERVs are underway (Ito et al., 2017), but the regulatory relationships in particular diseases have yet to be robustly defined. HERV dysregulation has been observed in chronic inflammatory and neurodegenerative diseases including amyotrophic lateral sclerosis (Douville and Nath, 2014), multiple sclerosis (Perron et al., 1997; Mameli et al., 2012), and schizophrenia (Frank et al., 2005).

There are at least two ways in which tau-mediated HERV activation may play a role in the development of Alzheimer’s disease: through the upregulation of HERVs or via the mobilization of HERVs. Tau may be involved in chromatin remodeling around HERV sequences, making regions in which HERVs are found increasingly transcriptionally accessible (Sun et al., 2018). HERV-derived nucleic acids, especially double-stranded DNA — which is a highly immunogenic pathogen-associated molecular pattern — may trigger the activation of innate immunity, as may HERV-derived proteins such as *env* which share structural similarity to extant viral proteins. In multiple sclerosis, HERVs have been shown to trigger an immune response *in vitro* (Perron et al., 2001), perhaps, as has been suggested, through the production of proinflammatory cytokines via the engagement of CD14/TLR4. In amyotrophic lateral sclerosis, proinflammatory stimuli seem to activate HERVs which, in turn, produce additional pro-inflammatory stimuli, hinting at a possible positive feedback mechanism in which epigenetically de-repressed HERVs amplify abnormal immune responses. Not only might the transcription of HERVs themselves be involved in pathogenesis, but the corresponding accessibility of HERV sequences may allow for more frequent and persistent presence of transcriptomic machinery at these loci and facilitate the transcription of nearby genes. The structure of prototypical HERVs – in which long terminal repeat regions flank viral proteins *gag*, *pro*, *pol* and *env* – allow these sequences to serve as active promoters for flanking sequences. The constituent loss of homeostatic balance that arises from this upregulation of HERV-proximal genes may contribute in some way to the Alzheimer’s disease phenotype.

Recent studies have shed light on the possible role of the HERV-K and HERV-W *env* genes in neurodegenerative diseases, including Alzheimer’s disease (Antony et al., 2004; Perron et al., 2005; Li et al., 2015; Ibba et al., 2018). Human toll-like receptor (TLR) 8 is responsive to a GUUGUGU motif found within the *env* gene of some HERV-K(HML-2) elements. Activation of hTLR8 upon binding to the *env* gene induces the canonical TLR pathway, ultimately leading to neuronal apoptosis, increased microglia, and the release of proinflammatory molecules including interferon. Such a process could contribute to phenotypic changes characteristic of Alzheimer’s disease. Analysis of transcriptomic data from brain samples pointed to a correlation between upregulated HERV-K and TLR8 expression in the temporal cortex of Alzheimer’s disease patients compared to age-matched controls (Dembny et al., 2020).

The role of HERVs in Alzheimer’s disease has been explored at least since the early 2000’s (Johnston et al., 2001) but, until now, studies have failed to quantify HERVs in both a high-throughput and locus-specific manner. Telescope is a tool that uses Bayesian reassignment to accurately classify HERV expression from RNA-Seq data (Bendall et al., 2019). Critically, telescope differs from other tools because it is able to estimate expression at locus-specific insertions rather than at the broad, subfamily level. Such accuracy allows us, for the first time, to comprehensively probe the expression of HERVs in Alzheimer’s disease.

## 2. Materials and methods

### 2.1. RNA-Seq datasets

Here, we employ the use of three datasets. The first is from a heretofore unpublished cohort from Washington University, henceforth known as the WashU data. The study has been approved by the Institutional Review Board (Approval number: 201109148). Description of the data and raw data can be found at NIAGADS, dataset #00038.^1^ For this cohort RNA was extracted from the Parietal cortex and RNA-Seq was generated using a Ribo-zero library with 30 million 150 × 2 reads (Del-Aguila et al., 2019; Dube et al., 2019; Li et al., 2020).

Participants in this study have provided written consent for providing their samples and clinical and demographic metadata. The other two datasets are publicly available via SRA: PRJEB28518 and PRJNA670209 (Nativio et al., 2020). PRJEB28518 used samples collected from post-mortem human brain samples collected at Seoul National University. PRJNA670209 used samples from frozen postmortem brain tissue from the Center for Neurodegenerative Disease Research brain bank (at UPenn) from both young and old patients sequenced on a NextSeq 500 machine.

### 2.2. RNA-Seq HERV identification and expression

Here, we use telescope (Bendall et al., 2019) to perform locus specific HERV quantification from RNA-Seq data. Telescope deals with the ambiguous mapping of repetitive HERV sequences by employing a Bayesian mixture model and expectation-maximization algorithm to reassign ambiguously mapped RNA-Seq fragments to the most likely locus of origin, thereby facilitating accurate, locus-specific HERV quantification. Our software pipeline uses fastQC (Andrews, 2010) to check for sequence quality, Trimmomatic (Bolger et al., 2014) to trim reads, then Bowtie2 (Langmead and Salzberg, 2012) to align reads to the Hg38 reference genome (GRCh38_no_alt_analysis_set_GCA_000001405.15) using the very-sensitive-local setting and allowing for a maximum of 100 alignments per read (–very-sensitive-local -k 100 –score-min L, 0, 1.6). The alignment files generated by Bowtie2 are fed into telescope which then uses the aforementioned Bayesian reassignment using up to 200 iterations of an expectation-maximization algorithm which has been modified to identify transposable elements (TEs) (–max_iter 200 –theta_prior 200000). With telescope, TEs are inferred when the hallmark genomic signatures of such elements are identified, including 5′ and 3′ long terminal repeats (LTRs) with an open reading frame between, thus inferring a functional TE. A total of 14,968 HERVs have thus been identified in the human genome and their annotation can be found at https://github.com/mlbendall/telescope_annotation_db/tree/master/builds/retro.hg38.v1. The output generated by telescope is a table of TEs (labeled by chromosomal location) and their expression as count values which are used in subsequent, downstream analyses.

### 2.3. RNA-Seq host gene expression

Host gene expression analysis was performed using fastQC for quality control, Trimmomatic for trimming of adapters and low-quality sequences, STAR (Dobin et al., 2013) for alignment, and FeatureCounts (Liao et al., 2014) for enumeration of transcripts.

### 2.4. Integrated gene and HERV expression

Arboreto (Moerman et al., 2019) was used to identify gene regulatory networks; specifically, it was used to identify those transcripts which show a high degree of correlation to each other. Variance-stabilization transformed counts of gene and HERV transcripts were used as input to Arboreto. The output was filtered to show those associations that existed between genes and HERVs (excluding HERV-HERV associations and gene-gene associations). Top associations, ranked by feature importance, were visualized in R using ggplot2.

### 2.5. Differential expression analysis

To account for sex-specific variance, genes that map to the Y chromosome were removed from the raw data. Batch effects associated with the provenance of the three datasets were removed using the ComBat-Seq function of the sva package in R v3.42.0 (Leek et al., 2012). Elements with low and consistent expression (which likely generate an uninformative signal and inflate adjusted *p*-values) were removed with the R package HTS Filter v1.34.0 which uses a Jaccard index filter to dynamically derive cutoff values to screen out uninformative transcriptomic data (Rau et al., 2013). Differential expression analysis comparing Alzheimer’s data to normal samples was performed with DESeq2 v1.34.0 using a significance cutoff of *p* = 0.05 and condition (Alzheimer’s or normal brain tissue) as factors in the DESeq model (Love et al., 2014). The R package ggplot2 v3.3.5 (Wickham, 2016) was used to visualize volcano plots, and the circlize v0.4.14 (Gu et al., 2014) package was used to visualize circos plots of differential HERV expression.

### 2.6. Proximal gene set enrichment and pathway analysis

Human endogenous retroviruses (HERVs) have been proposed as regulators that act on proximally-located genes (Buzdin et al., 2006; Rebollo et al., 2012; Sundaram et al., 2014; Yu et al., 2023). With this in mind, we sought to perform gene set enrichment analysis of those genes which were in the genomic neighborhood of the differentially expressed HERVs that were revealed in our analysis. Genes which flank or are intersected by HERVs have been documented for our telescope HERV annotation, available here: https://github.com/liniguez/telescope_metaannotations. Using an R script, differentially expressed HERVs were programmatically mapped to their closest upstream, downstream, and, in relevant cases, intersecting genes. The coefficient values associated with HERVs were assigned to their flaking/intersecting genes and these values were used as an input to omePath,^2^ a generic tool for pathway enrichment analysis that allows users to calculate importance scores for omics features (i.e., expression of genes and HERVs) appropriate for their study design (e.g., adjusting for multivariable testing and confounding factors). Here, we use omePath with a gene ontology (GO) biological process reference database (The Gene Ontology Consortium, 2019) for mapping omics features to pathways. omePath identifies which pathways have significant associations with the underlying features by performing statistical tests using the feature scores in the pathways against all ranks to calculate a *p*-value and false discovery rate (FDR) for hypothesis testing.

## 3. Results

In this study, we use three datasets, two are publicly available and the third we publish as part of this paper (Table 1). We report that our samples had a median read count of ∼30 million and the median sample had 150,000 reads map to HERV sequences (Supplementary Figure 1). The HERV families with the most mapped reads were HERVL and ERVLE.

**TABLE 1:** Brief description of datasets.

Dataset name	Number of AD patients	Number of healthy controls	Min # of reads	Median # of reads
WashU	81	17	3,096,385	29,422,202
PRJEB28518	10	10	21,645,208	44,218,524
PRJNA670209	12	18	26,043,105	38,416,313

The table outlines the number of patients in each cohort along with the minimum and median number of sequencing reads from each dataset.

**FIGURE 1:**
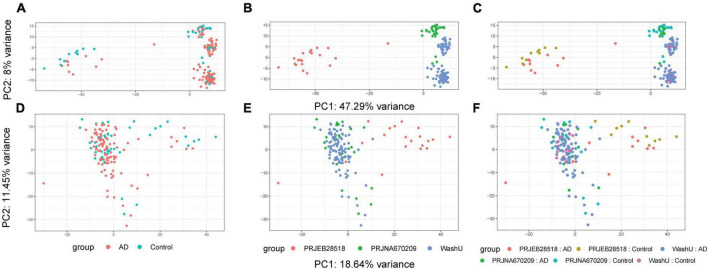
Results of batch effect correction on data structure shown via PCA. Before and after batch correction. Prior to batch correction using ComBat-Seq, much of the variance could be attributed to the provenance of the data sets. **(A)** PCA plot before batch effect correction colored by group. **(B)** PCA plot before batch effect correction colored by source. **(C)** PCA plot before batch effect correction colored by group and source. **(D)** PCA plot after batch effect correction colored by group. **(E)** PCA plot after batch effect correction colored by source. **(F)** PCA plot before batch effect correction colored by group and source.

Due to the different provenance of our three datasets, we first sought to perform batch effect correction using ComBat-Seq on the count values of our HERV expression matrices. An initial PCA (principal component analysis) was performed before batch effect correction and, as expected, samples were clearly separated–not only by disease status but, more worryingly, by provenance (Figures 1A–C). After performing batch effect correction, samples were re-analyzed using the same PCA method and data provenance was no longer such a prominent factor (Figures 1D–F). With our count values thus corrected across our three datasets, we proceeded to the next analysis step.

**FIGURE 2:**
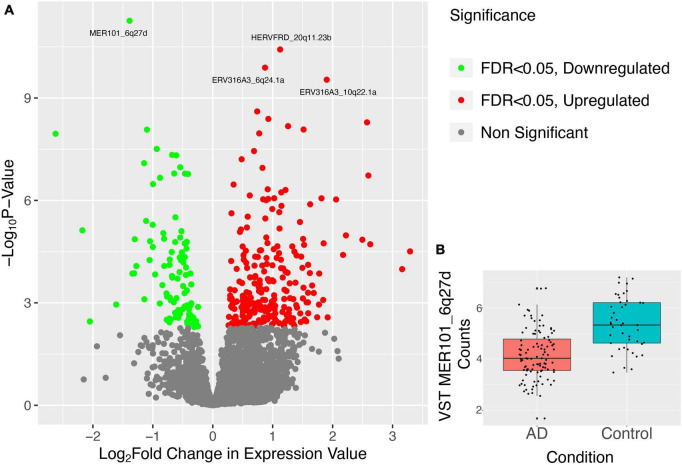
Results of differential expression analysis of HERVs in combined Alzheimer’s dataset. **(A)** Points in the volcano plot are individual HERV loci, their *X*-axis position represents their log_2_ fold change and their *Y*-axis position represents the –log_10_ of the *p*-value. Thus, HERVs higher on the plane and further to the edges represent more significantly aberrantly expressed HERVs where the bright green HERVs are those which have a FDR < 0.05 and are downregulated and the bright red HERVs are those which have a FDR < 0.05 and are upregulated. The top 4 most DE HERVs, ranked by *p*-value, are labeled. **(B)** Variance-stabilizing transformed counts of the most DE HERV, MER101_6q27d, are shown in box plots separated into two categories, AD patients and healthy controls.

Telescope quantified 13,666 distinct HERV loci expressed across the patients in our dataset. We performed differential expression using DESeq2 analysis on these HERVs between healthy and diseased patients. Doing so, we identified 698 HERVs that were differentially expressed (FDR adjusted *p*-value < 0.2) between Alzheimer’s and normal brain samples. Of the 698 elements, 187 were downregulated and 511 were upregulated (Figure 2).

**FIGURE 3:**
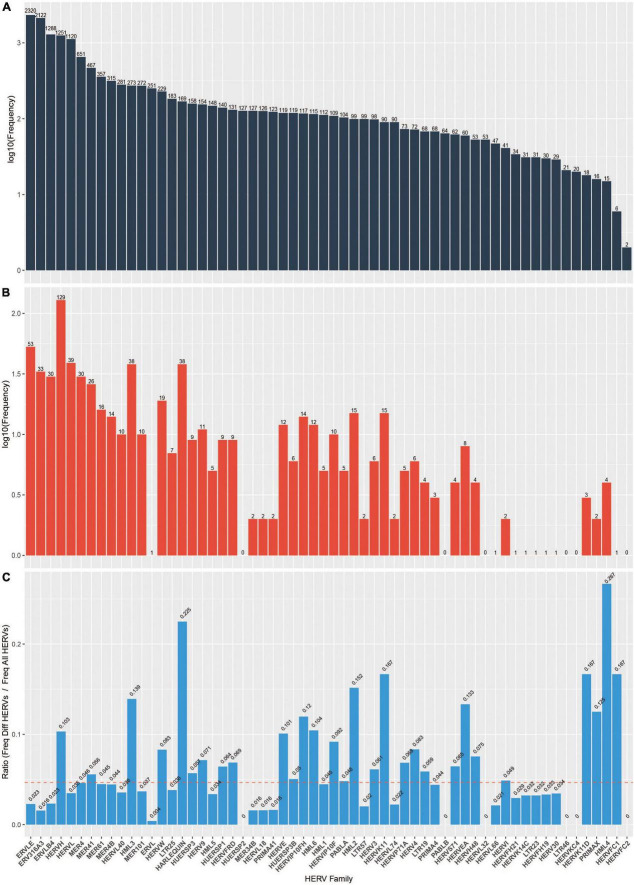
HERV frequency by family. **(A)** log_10_ frequency of HERV families in the database **(B)** log_10_ frequency of HERV families among the DE HERVs in our analysis **(C)** Ratio of frequency of DE HERVs in our analysis divided by frequency in the database. Dashed red line indicates the expected ratio (i.e., total number of HERVs in the database divided by total number of DE HERVs).

As previously mentioned, HERVs can be divided into functional families. We sought to identify which families (if any) were disproportionately represented among the 698 significantly differentially expressed HERVs we identified. Indeed, members of HERV families including HML4, HARLEQUIN, HERVFC1, HERVK11D, HERVK11 are over-represented in the differentially expressed HERVs (Figure 3).

Because HERVs are dispersed throughout the human genome, we sought to identify whether particular genomic regions harbored a disproportionate number of differentially expressed HERVs. Significantly differentially expressed HERVs were scattered throughout the genome and showed no obvious pattern of chromosomal over-representation (Supplementary Figure 2).

**FIGURE 4:**
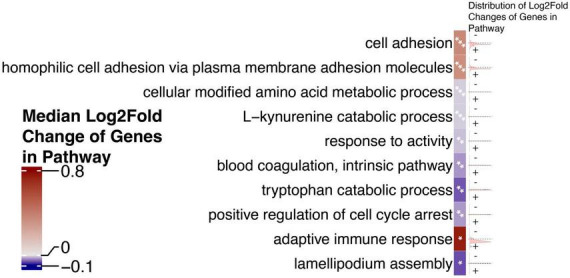
Gene set enrichment analysis of genes proximal to differentially expressed HERVs reveals dysregulated pathways that may be at play in AD. The top 10 most significant pathways, ranked by *p*-value are shown. Cells which contain one asterisk (*) represent pathways with *p*-values less than 0.05 but greater than 0.005. Cells which contain two asterisks (**) represent pathways with *p*-values less than 0.005 but greater than 0.001. Cells which contain three asterisks (***) represent pathways with *p*-values less than 0.001. Cells are annotated on the right with density plots showing the distribution of the log_2_ fold changes of genes in a given pathway. Cells are colored by the median log_2_ fold change of genes in the pathway to indicate the general directionality of the pathway in AD.

Because HERVs are hypothesized to act as transcriptional regulators, especially for proximal genes, we chose to investigate whether HERVs play a role in activating genes that drive Alzheimer’s disease. We queried the role of neighboring genes using a gene enrichment analysis. We identify pathways that are significantly enriched using this set of genes proximal to dysregulated HERVs (Figure 4).

**FIGURE 5:**
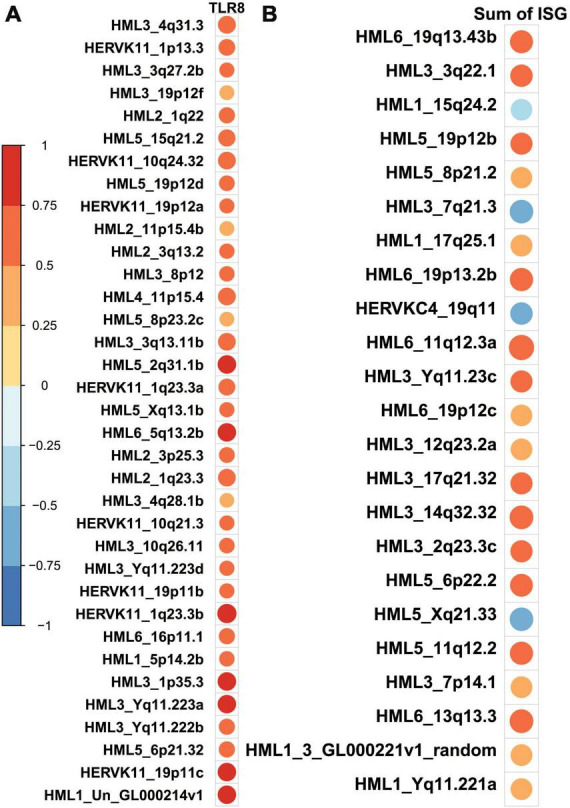
HERV-K loci which are significantly correlated with gene expression of interest tend to show positive correlations. Cells show circles whose size and color are indicative of the Pearson correlation coefficient associated with a correlation test between the transcript counts of the transcripts and HERV-K loci. Larger, redder circles indicate correlation coefficients that approach 1. Larger, bluer circles indicate correlation coefficients that approach –1. Only significant associations are shown in order to save space; a full depiction of the correlation between all HERV-K loci and TLR8 is given in the Supplementary material. **(A)** HERV-K loci which are significantly correlated with TLR8 expression all show a positive direction of such a correlation. **(B)** HERV-K loci which are significantly correlated with a sum of the expression of interferon-stimulated genes (ISG) mostly show a positive direction of such a correlation–19/23 correlation values are positive.

To further explore patterns identified in previous research that show HERVK molecules as playing a particularly important role in modulating biological processes in Alzheimer’s patients, we conducted a correlation analysis between the quantification of HERVK molecules and the expression of TLR-8 as well as the summed expression of genes that comprise an interferon signature (Catalina et al., 2019; Supplementary Table 2). We note that HERVK expression tends to positively correlate with expression of both TLR-8 and interferon signature genes (Figure 5) but that only a subset of HERVK loci are significantly correlated with either (Supplementary Figure 3). We also report that significant correlation patterns with TLR-8 and HERVK expression are absent at the family level. Specifically, the sum of neither HML1, HML2, HML3, HML4, HML5, HML6, HERVK11, HERVK11D, nor HERVKC4 elements, each families within the superfamily of HERVK, are significantly correlated with TLR-8 expression (Supplementary Figure 4).

**FIGURE 6:**
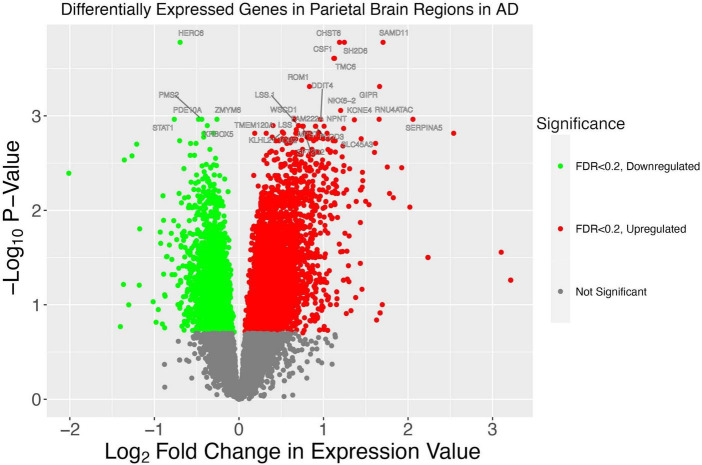
A volcano plot illustrates patterns of gene dysregulation in AD. On the *y*-axis the –log_10_*P*-value calculated using DESeq2 is shown. On the *x*-axis, the log_2_ fold change in expression value is shown. Each dot represents a gene. A handful of the genes with the most extreme *p*-values are shown. Red dots lie to the right of the *x* = 0 asymptote and represent genes with a greater expression in AD. Green dots lie to the left of the *x* = 0 asymptote and represent genes which have decreased expression in AD. All dots below the line of –log_10_(0.2) are colored gray and represent genes which have a statistically insignificant change between conditions.

**FIGURE 7:**
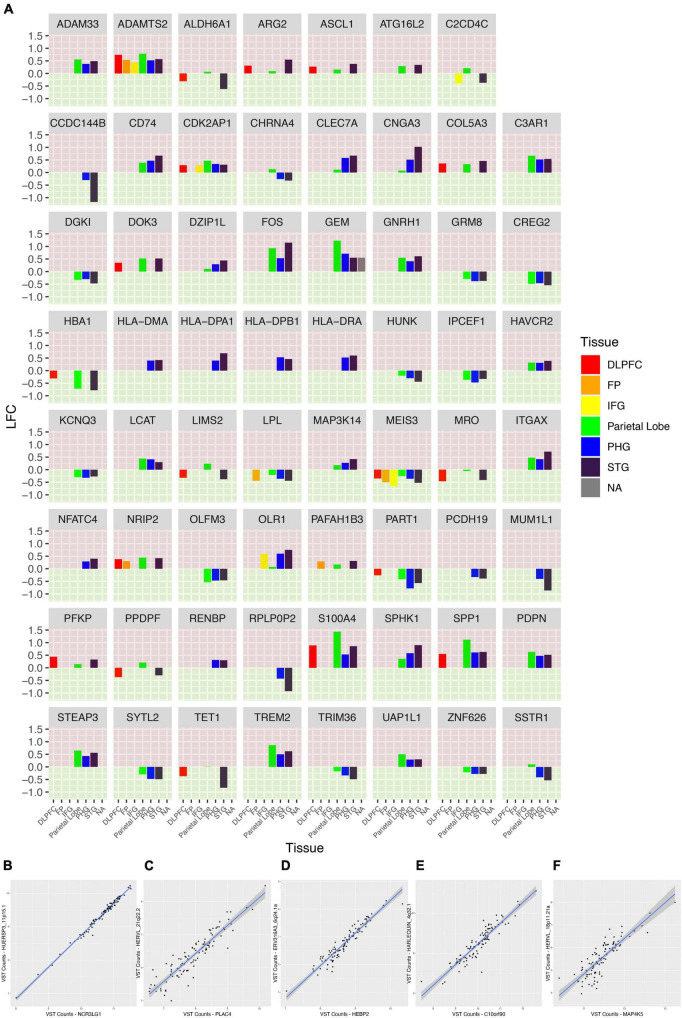
A comparison of the fold change of genes across brain regions in AD. **(A)** Faceted plot is shown in which each panel represents a gene. Along the *x*-axis are brain regions and the fold change of the gene in patients with AD is expressed by the height of the bar. Each panel is colored into green and red regions to make it clear which direction the bars face, and, in turn, the corresponding fold change of the gene. The upper portion of each panel is colored red; bars which stretch into this region represent genes with a positive log fold change in AD. The lower portion of each panel is colored green; bars which stretch into this region represent genes with a negative log fold change in AD. Each tissue is also given its own color; some genes were not expressed in some tissues or not associated with a significant log fold change and, as a consequence, not every panel contains data for every tissue. Largely the patterns of log fold change remain consistent across tissues but there are exceptions in which genes show a different pattern of expression in the parietal lobe compared to other tissues. **(B–F)** Variance-stabilizing transformed counts of top 5 most-associated genes and HERVs as calculated by Arboreto.

We also sought to characterize the gene expression of the samples from our WashU RNA-Seq dataset. We report 1,514 upregulated genes in AD (FDR < 0.05) and 685 downregulated genes in AD (FDR < 0.05) (Figure 6). In order to compare the differentially expressed genes in our study — in which tissue was collected from the parietal lobe — with the results of other studies conducted in AD across brain regions, we obtained log fold change values from genes presented in a recent study (Figure 7A). Additionally, we used Arboreto to find HERVs that show the highest associations with DE genes and present scatter plots of the 5 strongest associations, ranked by feature importance (Figures 7B–F).

**FIGURE 8:**
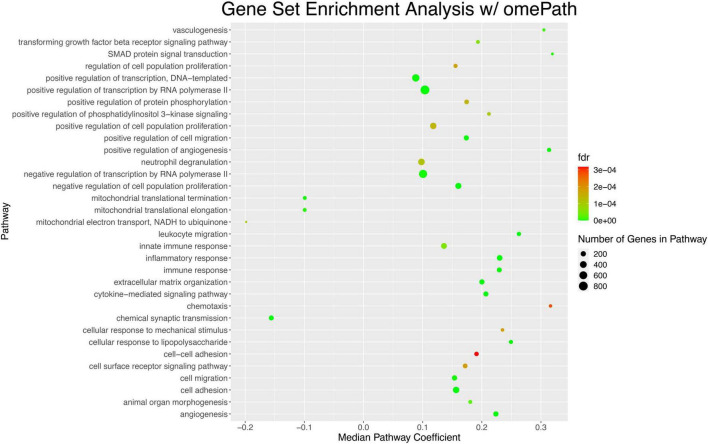
Gene set enrichment analysis of DE genes in AD highlights pathways that are significantly dysregulated in the parietal lobe. The top 33 pathways sorted by lowest *p*-value are shown. Each dot represents a pathway; the position of the dot on the *x*-axis represents the coefficient of the median gene in that pathway. The color of the gene, on a color scale from red to green, represents an increasingly more significant *p*-value associated with the dysregulation of the pathway. The size of the dot indicates the number of genes in the pathway; in this case pathways ranged from ∼200 genes to ∼800 genes.

Finally, we conducted gene set enrichment analysis and identify chemotaxis and cell-cell adhesion as pathways that were the most statistically significantly enriched (Figure 8).

## 4. Discussion

In this analysis, we present locus-specific differential HERV expression patterns in Alzheimer’s disease compared to normal brain samples. After filtering uninformative elements, 698 HERVs were found to be differentially expressed between Alzheimer’s and normal brain samples. Of the 698 elements, 511 were upregulated and 187 were downregulated. Of the 62 families included in the analysis, several had more differentially expressed elements than would have been expected statistically. These families include HERV-K, HERV-W, HML3, HML2, HML4, and HML6, among others (Figure 3).

After identifying elements of interest, we conducted a differential gene expression analysis to assess dysregulation of elements proximal to individual HERV loci. We assessed the relationship between HERV-K and TLR8 expression, two elements that may work in tandem to cause neurodegeneration (Dembny et al., 2020). The association between TLR8 and each of the nine HERV-K families were found to be statistically insignificant (Supplementary Figure 2). However, when looking at individual loci, a number of elements had statistically significant Pearson correlation coefficients approaching one (Figure 6). Of note, all of the significant associations were positive. Similarly strong trends were observed by correlating HERV-K expression with the summed expression of a set of interferon signature genes. These results not only corroborate the findings of previous studies, they also highlight the benefit of looking beyond the family level of HERVs to specific transposable element insertions.

Next, gene set enrichment analysis of genes proximal to differentially expressed HERVs reveals dysregulated pathways that may be at play in AD. The pathways with the most statistically significant coefficient values were both linked to cell adhesion process, while the pathway with the largest absolute value of the coefficient was linked to the cell cycle. Disruptions to the cell cycle of neurons in the AD brain have been noted for several decades. As early as 1997, McShea et al. (1997) identified a number of dysregulated cell cycle mediators, such as cell-division cycle 2 (cdc2), cyclin B1 kinase, and cyclin-dependent kinase-4 (Cdk4) (Vincent et al., 1997). Soon after, evidence emerged that neurons in AD patients likely re-enter an ultimately lethal cell cycle (Yang et al., 2001), a cellular process that is now considered an early manifestation of neurodegenerative disease (Denechaud et al., 2023).

Our proximal gene analysis indicated that a member of the transmembrane (*TMEM*) protein family, *TMEM179* (ENSG00000258986), had the highest coefficient value at 2.623108848. The *TMEM* family is responsible for fulfilling a wide variety of important physiological functions such as mounting an immune response (Dodeller et al., 2008), collagen secretion (Zhang et al., 2020), dendritic lysosomal trafficking (Zhang et al., 2020), and smooth muscle contraction (Thomas-Gatewood et al., 2011), among other roles. The differential expression of the *TMEM* family has also been noted in several types of cancers (Schmit and Michiels, 2018). Of the expansive list of *TMEM* proteins, *TMEM179* is less frequently a subject of study. The sequence is located on human 14q32.33, which has previously been identified as a potential evolutionary breakpoint (Ruiz-Herrera et al., 2006; Longo et al., 2009). This protein has recently been identified as one of eight probable modulators of optic nerve necrosis in mice (Stiemke et al., 2020). It has also been demonstrated that *TMEM179* is expressed in oligodendrocyte precursor cells (OPCs), astrocytes, and neurons; and that it is found in the cortex, hippocampus, and hypothalamus of mice (He et al., 2021). However, the primary focus of the study was investigating the neurotoxic effects of arsenic and the protective effects of N-acetyl-cysteine (NAC) on OPCs. The investigators found that *TMEM179* was likely involved in modulating both neurotoxic and protective effects in OPCs, in addition to being involved in mitochondrial maintenance and apoptotic pathways (He et al., 2021). Notably, *TMEM179* was thought to inhibit apoptosis via suppression of protein kinase C β (PKCβ) (He et al., 2021), which is one of a handful of PKC isoforms often referred to as “memory kinases” for their role in transmembrane signal conduction (Sun and Alkon, 2014). Mice deficient in PKCβ also demonstrate deficits in cued conditioning and learning (Weeber et al., 2000). Most relevant though is the purported role of PKCβ in AD pathology. Studies have shown that when PKCβ fails to translocate to the plasma membrane, it can hyperphosphorylate tau (Gerschütz et al., 2013).

Accumulation of detritus could also be attributed to dysregulation of the *PSMB1* gene—which was found to have a log_2_ fold change value of 1.388246983. This gene encodes the β6 subunit of a proteasome beta-type family which in turn forms the core of the 20S proteasome. Proteasomes are key members of the ubiquitin–proteasome system (UPS) which is responsible for degradation of intracellular damaged, mutant, misfolded, or extraneous proteins (Glickman and Ciechanover, 2002). Critically, reduced UPS function has been noted throughout much of early Alzheimer’s literature and could be linked to the accumulation of tau seen in the AD brain (López Salon et al., 2000; Keller et al., 2001). The 20S proteasome in particular is affected by this aberrant control of UPS function observed in AD. The phosphorylation and subsequent deactivation of nuclear factor erythroid 2-like factor 2 (Nrf2) in AD patients prevents the increased expression of the 20S proteasome (Ramsey et al., 2007; Pickering et al., 2012), which dwindles the already diminishing pool of competent proteasomes. While it is worth noting that researchers are still unclear whether defective proteasome function causes AD or if it arises as a consequence of the disease and simply exacerbates it (Lee et al., 2013), the increased expression of the *PSMB1* gene could still be an indication of the body attempting to compensate for reduced UPS activity.

Other overexpressed genes found in the analysis have less tangential relations to neurodegenerative changes. One such gene was *BASP1*, which had a log_2_ fold change value of 1.30198558. This gene plays a critical role in neurodevelopment, synaptic function, and nerve regeneration. More specifically, it is involved in enhanced neurite outgrowth (Korshunova et al., 2008), has implications for neurotransmission, synaptic plasticity, and information storage (Yamamoto et al., 1997; Han et al., 2013), and axonal regeneration after injury. Notably, recent studies have shown that levels of *BASP1* are decreased in AD patients (Musunuri et al., 2014), and the protein often demonstrates decreased phosphorylation in the temporal lobes compared to non-AD subjects (Tagawa et al., 2015).

A number of genes with both positive and negative correlation coefficients are classified as long non-coding RNAs (lncRNAs) and pseudogenes. While these specific elements had little to no coverage in literature, lncRNAs in general have been implicated in a number of human diseases, including Alzheimer’s. Although lncRNAs are not capable of forming functional proteins, they nonetheless play a crucial role in mediating the expression of neighboring protein-coding genes through chromatin modifications, transcriptional regulation, and post-transcriptional regulation (Mercer et al., 2009; Derrien et al., 2012; Marchese et al., 2017). Due to their regulatory roles, genomic rearrangements with lncRNA loci have been linked to cancers, neurological and cardiovascular disorders, and infectious or inflammatory disease (Esteller, 2011; Wapinski and Chang, 2011; DiStefano, 2018; Aznaourova et al., 2020). In the past decade, lncRNAs have been increasingly linked to AD pathology, with a number of elements having been identified as potential biomarkers in the blood (Kurt et al., 2020), plasma (Feng et al., 2018), extracellular vesicles (Straten et al., 2009; Wang et al., 2020), and cerebrospinal fluid (Straten et al., 2009; Wang et al., 2020). These identified elements (Supplementary Table 1) may also serve as biomarkers or play a role in AD pathology.

## 5. Conclusion

A total of 698 HERVs were identified as being significantly differentially expressed in Alzheimer’s disease vs. healthy controls. These HERVs are physically proximal to genes in cell adhesion and immune response pathways. HERVs in the HERV-K superfamily are overrepresented among those differentially expressed and many loci are correlated with innate immune activation.

Further studies will be necessary to confirm the role of specific HERV loci in innate immunity and cell adhesion in neurodegenerative diseases like Alzheimer’s Disease. Both *in vitro* and *in vivo* experimentation are warranted. Future studies may also probe the expression of other transposable elements or perform binding experiments to show to what degree certain HERV transcripts or proteins are immunogenic in AD.

## Data availability statement

The data is available at https://www.niagads.org/datasets/ng00083. Circular RNAs in Alzheimer’s disease brains – RNA-seq data. This dataset presents the results from processing ribosomal RNA (rRNA)-depleted RNA-seq data derived from human brain tissues donated by individuals with and without Alzheimer’s disease (AD). At the individual-level, it includes data for circular and linear RNA counts as well as technical, clinical, and neuropathological phenotypes. At the summary statistic-level, it includes discovery, replication, and meta-analysis circular RNA (circRNA) differential expression and AD-trait correlation results www.niagads.org.

## Ethics statement

The studies involving human participants were reviewed and approved by the Washington University Institutional Review Board. The patients/participants provided their written informed consent to participate in this study.

## Author contributions

TD, UR, and JS: investigation. CC, JK, and KC: conceptualization, resources, review and editing, supervision, and funding acquisition. TD, UR, JS, CC, JK, and KC: methodology. TD and UR: writing. All authors contributed to the manuscript and approved the submitted version.

## Footnotes

1
https://www.niagads.org/datasets/ng00083

2
https://github.com/omicseye/omepath

## Supplementary material

Supplementary Figure 1 Read counts in Wash-U dataset of 98 patients. **(a)** The total number of reads from each sample is shown. Each bar represents a particular sample and its height represents the number of total reads. **(b)** A summary boxplot is shown to represent the distribution of reads across our samples. **(c)** The number of reads from each sample that mapped to HERVs is shown. The order of samples from panel a is preserved. **(d)** A heatmap shows the number of HERVs across families that mapped from each sample. Darker shades of red indicate a higher number of mapped HERVs from a sample to a HERV family.

Supplementary Figure 2 Genomic location of HERVs expressed in Alzheimer’s disease. DE HERV loci are mapped to chromosomal locations and shown on a circos plot. The circos plot is a circular representation of the genome. The outermost ring is shaded according to cytogenetic bands with red bands corresponding to centromeres and black and gray bands corresponding to changes between bands and sub-bands that represent chromosomal structural features visible with a microscope. Chromosomes are labeled with text that surrounds this ring. The blue, dashed line in the center represents a log_2_ fold change of 0. Each dot represents a DE HERV in its corresponding chromosomal location, the color and distance from center are indicative of *p*-value and log_2_ fold change, respectively.

Supplementary Figure 3 Correlations between TLR-8 expression and expression of all HERV-K loci. Significant correlations have loci names written in red text.

Supplementary Figure 4 Correlation of sum of HERV-K expression across 9 HERV-K families and TLR8 expression.
