# Supplementary material for: Locus specific endogenous retroviral expression associated with Alzheimer’s disease
Source: Front Aging Neurosci. 2023 Jul 6;15:1186470. doi: 10.3389/fnagi.2023.1186470 (PMC10359044; doi:10.3389/fnagi.2023.1186470)
Supplement: Supplementary file 1 [file Table_1.docx]

| Gene | Coefficient |
| --- | --- |
| ENSG00000286966 | -3.29207719 |
| ENSG00000284294 | -3.160212785 |
| ENSG00000270425 | -2.627561428 |
| ENSG00000237506 | -2.173820675 |
| ENSG00000220326 | -2.026639097 |
| ENSG00000222659 | -2.026639097 |
| ENSG00000259126 | -1.917049257 |
| ENSG00000258487 | -1.917049257 |
| ENSG00000223368 | -1.872793220 |
| ENSG00000252482 | -1.849828649 |
| ENSG00000234811 | -1.849828649 |
| ENSG00000232155 | 1.275404722 |
| ENSG00000267084 | 1.312581627 |
| ENSG00000232964 | 1.611564386 |
| ENSG00000235817 | 1.611564386 |
| ENSG00000231995 | 1.932598364 |
| ENSG00000231212 | 1.932598364 |
| ENSG00000274020 | 2.050835255 |
| ENSG00000213159 | 2.623108848 |

**Supplementary Table 1** lncRNAs differentially expressed in Alzheimer’s Disease with their corresponding coefficients
