# Supplementary material for: Locus specific endogenous retroviral expression associated with Alzheimer’s disease
Source: Front Aging Neurosci. 2023 Jul 6;15:1186470. doi: 10.3389/fnagi.2023.1186470 (PMC10359044; doi:10.3389/fnagi.2023.1186470)
Supplement: Supplementary file 2 [file Table_2.docx]

| CXCL10 |
| --- |
| EDN1 |
| GLS |
| STAT2 |
| IDO1 |
| OAS3 |
| OAS1 |
| EPB41 |
| CXCL9 |
| LMNB1 |
| CDKN1A |
| MRPS15 |
| WARS |
| CCND2 |
| CFB |
| SP100 |
| JAK2 |
| ETV4 |
| CASP1 |
| LAP3 |
| STX11 |
| ATF3 |
| TAP2 |
| AIM2 |
| GBP2 |
| CD38 |
| MSR1 |
| IL15 |
| GCH1 |
| CCL8 |
| TNFSF10 |
| IL15RA |
| FAS |
| SERPING1 |
| GBP1 |
| PTCH1 |
| ACSL1 |
| TAP1 |
| APOL3 |
| F8 |
| GCNT1 |
| SOCS1 |
| CXCL11 |
| STAT1 |
| UBE2L6 |
| IFI44 |
| RGS1 |
| GADD45B |
| PSMB9 |
| NR3C1 |
| IFI27 |
| ECE1 |
| NMI |
| IFI35 |
| IFI16 |
| LEPR |
| LGALS2 |
| TGM1 |
| SPIB |
| INPPL1 |
| CCNA1 |
| COL3A1 |
| CASP5 |
| WT1 |
| TNFSF6 |
| EIF2AK2 |
| FAF1 |
| BCL7B |
| PRKRA |
| PDGFRL |
| JUP |
| MX1 |
| RGS6 |
| TRD |
| IKBKG |
| TFDP2 |
| MYD88 |
| KIF20B |
| PML |
| IFITM3 |
| CBWD1 |
| RBCK1 |
| UBE2S |
| MGLL |
| BRCA2 |
| DUSP7 |
| PDGFB |
| EIF2B1 |
| TRIM22 |
| GBAP1 |
| LY6E |
| LGALS3BP |
| HLA-DOA |
| NAMPT |
| PATJ |
| IRF7 |
| BST2 |
| LMO2 |
| SUPT3H |
| KRT8 |
| SP110 |
| DEFB1 |
| IFI6 |
| GUK1 |
| PKD2 |
| MAP2K5 |
| LGMN |
| AKAP2 |
| APOBEC3G |
| CD2AP |
| XAF1 |
| BARD1 |
| UNC93B1 |
| TRIM21 |
| BLVRA |
| DYSF |
| TRA2B |
| GPR161 |
| ATF5 |
| USP18 |
| GMPR |
| TLR7 |
| IFIT5 |
| UBA7 |
| NUB1 |
| ST3GAL5 |
| KCNA3 |
| IFIT1 |
| LAMP3 |
| DYNLT1 |
| CAD |
| CAMK2A |
| NFE2L3 |
| HS6ST1 |
| KPNB1 |
| MED1 |
| DUSP5 |
| OAS2 |
| SIT1 |
| PLSCR1 |
| SP140 |
| ENPP2 |
| IFITM1 |
| CXCR2 |
| LGALS9 |
| SCARB2 |
| EIF4ENIF1 |
| TCN2 |
| TRIM38 |
| CTSL |
| CHKA |
| AGT |
| CYP2J2 |
| PMAIP1 |
| ISG20 |
| FLNA |
| TNK2 |
| FGF1 |
| BAG1 |
| FUT4 |
| IFRD1 |
| FOXO1 |
| MCL1 |
| NUPR1 |
| HIST2H2AA3 |
| CCL7 |
| DSC2 |
| DLL1 |
| CNTN6 |
| TNFRSF11A |
| IL6 |
| TLR3 |
| APOBEC3B |
| TARBP1 |
| ADAR |
| RTP4 |
| SAT1 |
| TOR1B |
| MNDA |
| IRF2 |
| ISG15 |
| MX2 |
| HSP90AA1 |
| IL1RN |
| CBR1 |
| KLF6 |
| STAP1 |
| TRIM34 |
| CCL13 |
| IFITM2 |
| FTL |
| HBG2 |
| CASP10 |

**Supplementary Table 2** List of Interferon-Stimulated Genes
