# Supplementary figures and images for: Locus specific endogenous retroviral expression associated with Alzheimer’s disease
Source: Front Aging Neurosci. 2023 Jul 6;15:1186470. doi: 10.3389/fnagi.2023.1186470 (PMC10359044; doi:10.3389/fnagi.2023.1186470)

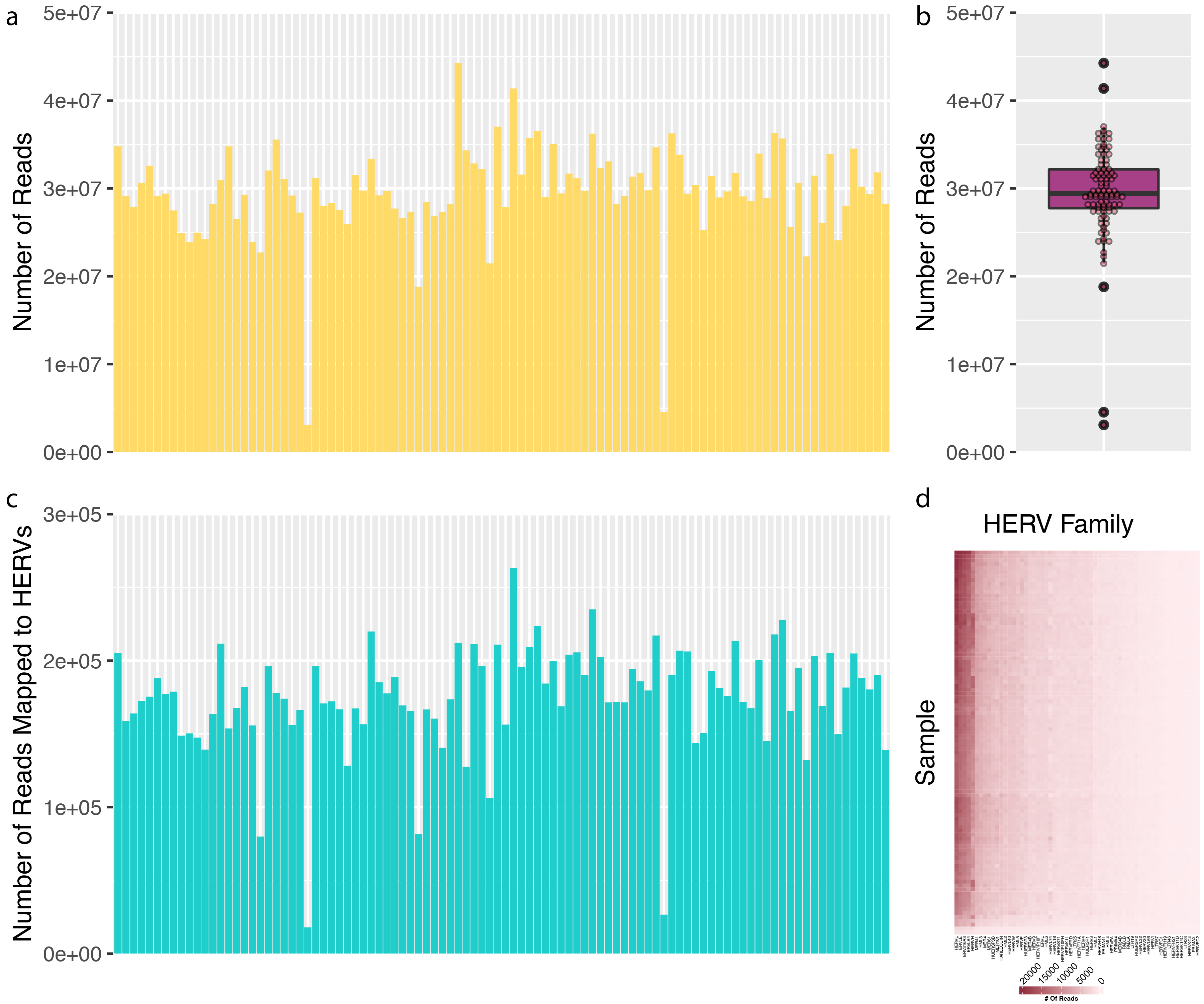

Supplement: Supplementary Figure 1 — Read counts in Wash-U dataset of 98 patients. (a) The total number of reads from each sample is shown. Each bar represents a particular sample and its height represents the number of total reads. (b) A summary boxplot is shown to represent the distribution of reads across our samples. (c) The number of reads from each sample that mapped to HERVs is shown. The order of samples from panel a is preserved. (d) A heatmap shows the number of HERVs across families that mapped from each sample. Darker shades of red indicate a higher number of mapped HERVs from a sample to a HERV family. [file Image_1.JPEG]

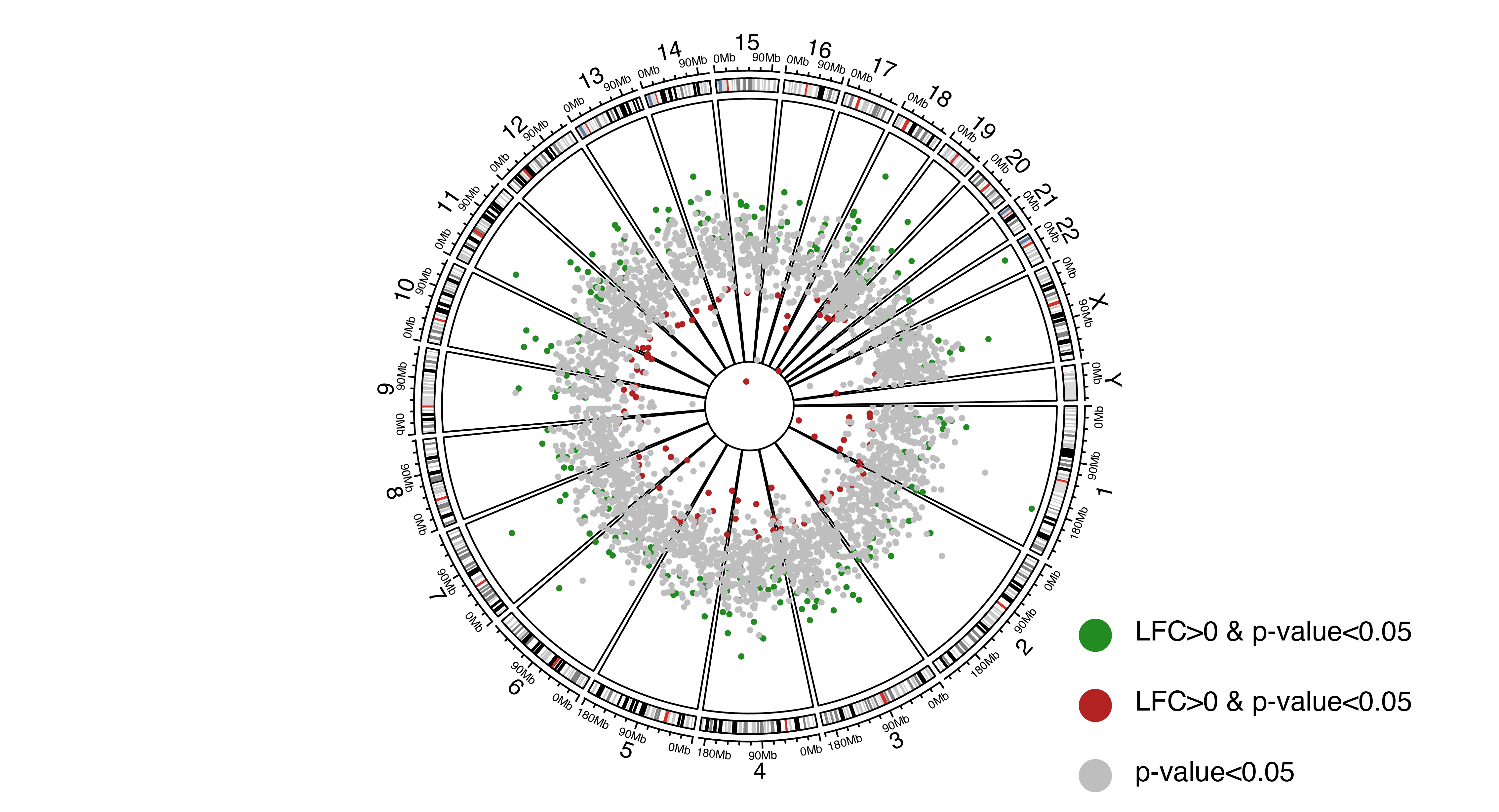

Supplement: Supplementary Figure 2 — Genomic location of HERVs expressed in Alzheimer’s disease. DE HERV loci are mapped to chromosomal locations and shown on a circos plot. The circos plot is a circular representation of the genome. The outermost ring is shaded according to cytogenetic bands with red bands corresponding to centromeres and black and gray bands corresponding to changes between bands and sub-bands that represent chromosomal structural features visible with a microscope. Chromosomes are labeled with text that surrounds this ring. The blue, dashed line in the center represents a log2 fold change of 0. Each dot represents a DE HERV in its corresponding chromosomal location, the color and distance from center are indicative of p-value and log2 fold change, respectively. [file Image_2.JPEG]

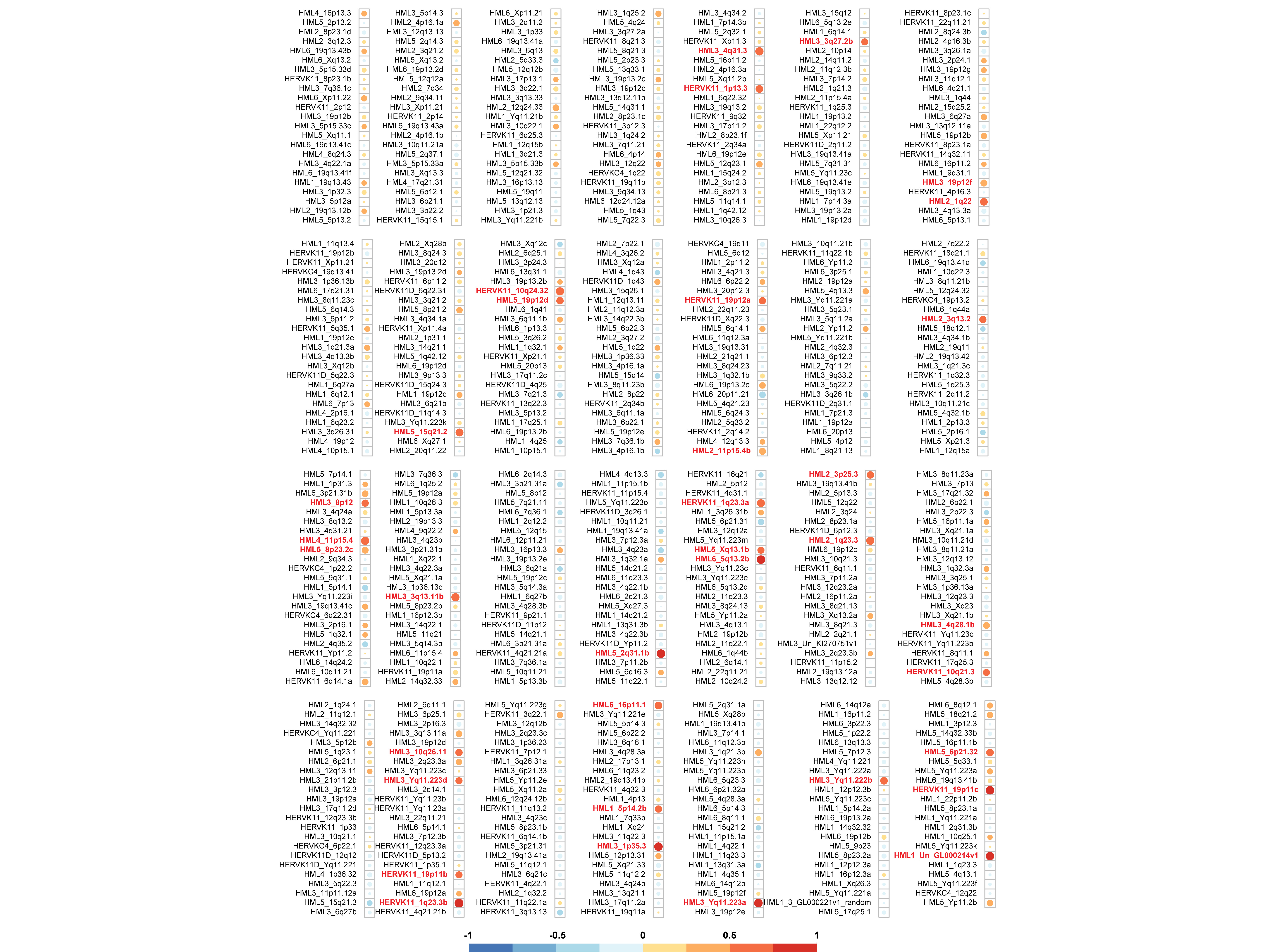

Supplement: Supplementary Figure 3 — Correlations between TLR-8 expression and expression of all HERV-K loci. Significant correlations have loci names written in red text. [file Image_3.JPEG]

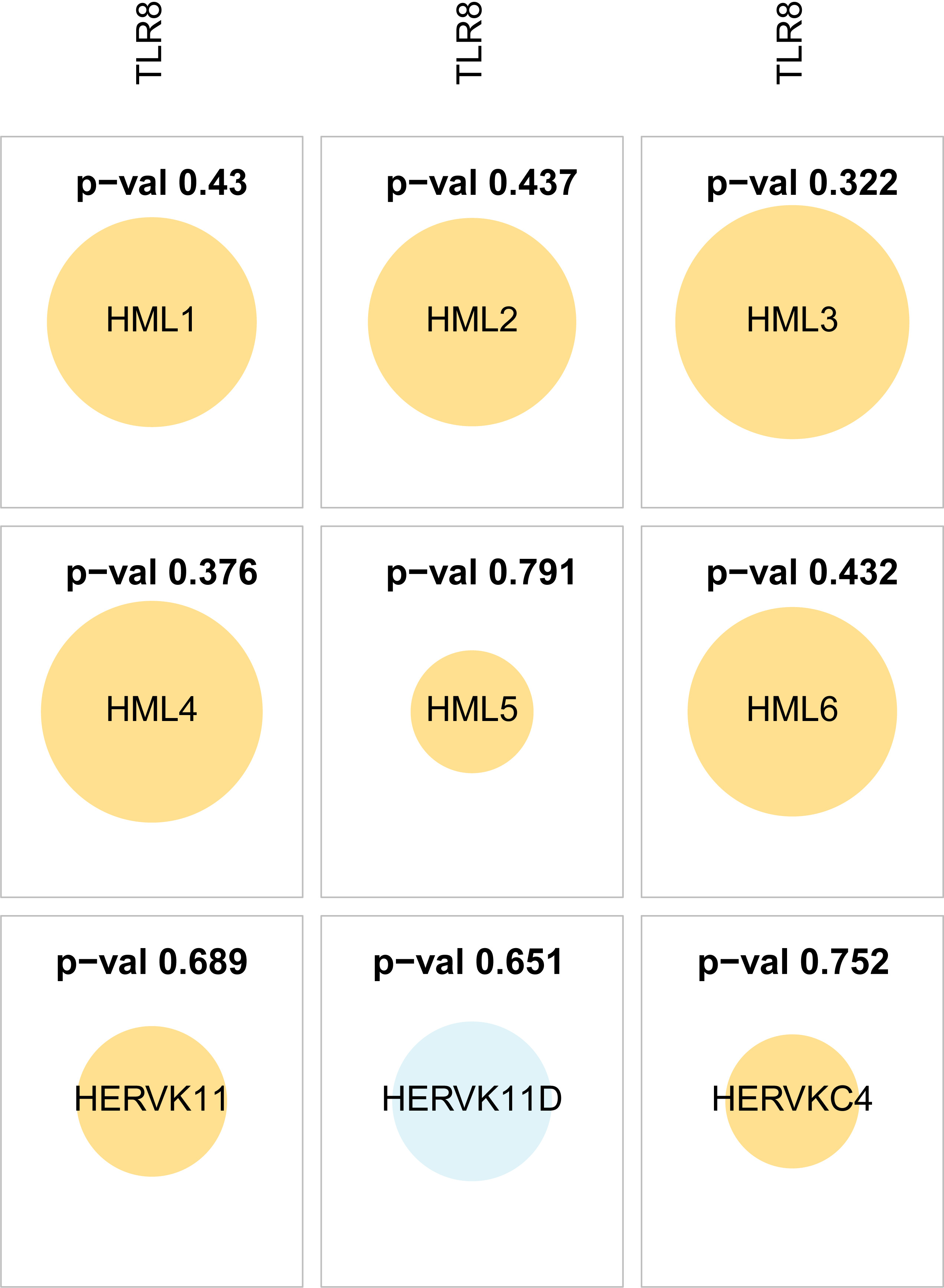

Supplement: Supplementary Figure 4 — Correlation of sum of HERV-K expression across 9 HERV-K families and TLR8 expression. [file Image_4.JPEG]
